# *Rubus* Capped Zinc Oxide Nanoparticles Induce Apoptosis in MCF-7 Breast Cancer Cells

**DOI:** 10.3390/molecules27206862

**Published:** 2022-10-13

**Authors:** Blassan P. George, Naresh K. Rajendran, Nicolette N. Houreld, Heidi Abrahamse

**Affiliations:** Laser Research Centre, University of Johannesburg, Johannesburg 2028, South Africa

**Keywords:** *Rubus*, zinc oxide, nanoparticles, MCF-7, apoptosis, caspases

## Abstract

*Rubus fairholmianus* (RF) has widely been used to treat various ailments, including pain, diabetes, and cancer. Zinc oxide nanoparticles (ZnO NPs) have drawn attention in modern healthcare applications. Hence, we designed this study to synthesize zinc oxide (ZnO) nanoparticles using *R. fairholmianus* root extract to investigate its synergistic cytotoxic effect on MCF-7 cells and explore the possible cell death mechanism. ZnO NPs were synthesized via green synthesis using *R. fairholmianus* root extract, and the effect on MCF-7 cells was determined by looking at cellular morphology, proliferation, cytotoxicity, apoptosis, and reactive oxygen species (ROS). The results showed that cellular proliferation was reduced following treatment with *R. fairholmianus* capped zinc oxide nanoparticles (RFZnO NPs), while cytotoxicity and ROS were increased. There was also an increase in apoptosis as indicated by the significant increase in cytoplasmic cytochrome c and caspase 3/7 (markers of apoptosis), as well as increased levels of pro-apoptotic proteins (p53, Bax) and decreased levels of anti-apoptotic protein (Bcl-2). In conclusion, these results showed that RFZnO NPs induce apoptosis in breast cancer cells via a mitochondria-mediated caspase-dependent apoptotic pathway and suggest the use of acetone root extract of *R. fairholmianus* for the treatment of cancer-related ailments.

## 1. Introduction

Cancer is an abnormal and uncontrolled growth of cells resulting in an increased mass or lump of cells called tumors. Cancer is a serious health concern worldwide with significant morbidity and mortality [1]. Various well-established treatments are available to treat cancer, though the side effects and high cost make the fight against cancer difficult, particularly in developing countries. Chemotherapy is one of the effective cancer treatments, but it still displays minimal specificity and is limited by the severity of the side effects and toxicity toward healthy cells. It has become a challenge to find an effective therapy for cancer treatments [2]. Thus, traditional treatment methods need to be combined with modern drug delivery technology to enhance the treatment outcome and lessen the toxic side effects [3].

Nanomedicine is a promising anticancer niche area for developing novel diagnostic and therapeutic strategies. Green synthesis of nanoparticles (NPs) is a new strategy used for various biomedical applications. This is a cost-effective, eco-friendly, and biocompatible approach for the synthesis of NPs with varied biological properties [4]. Green synthesis uses plants and microbes and allows large-scale production of NPs without extra impurities, and such NPs show enhanced catalytic activity and limit the use of expensive and toxic chemically synthesized NPs [5]. Various plant parts (flower, leaf, stem, and root) have been used for the synthesis of NPs, and the phytocompounds in the plant extracts can act as both stabilizing and reducing agents during the process [6,7,8]. Recently, numerous metal oxide NPs such as copper (II) oxide (CuO), titanium (II) oxide (TiO), and zinc oxide (ZnO) have been synthesized through a green approach, and ZnO NPs have become popular in biomedical, molecular recognition, optics, and electronic applications due to the inexpensive, easy, and safe synthetic protocols [9,10,11]. The United States Food and Drug Administration (FDA) has approved ZnO as a safe metal oxide which showed semiconducting, catalytic, anti-inflammatory, and wound healing properties with a large band gap and high excitation binding energy [12,13,14]. Zinc is also an essential micronutrient in the human body and is vital for health and disease control, bone metabolism, and immune function [15,16,17]. Zinc-dependent proteins also play an important role in transcriptional regulation, cell death, and DNA repair [18,19].

The diverse genus *Rubus* includes over 750 species and is found on many continents [20]. *Rubus* species have been used in folk medicine due to their varied pharmacological properties. *R. fairholmianus* is also used for various ailments by tribal people [21,22,23]. We have reported the in vitro cytotoxic effects of *R. fairholmianus* on colorectal, breast, and lung cancer and melanoma cells [24,25,26]. The apoptotic efficacy of silver NPs conjugated with *R. fairholmianus* and the bioactive compounds of *R. fairholmianus* were studied on MCF-7 breast cancer cells [27,28]. We have recently reported on the biosynthesis of ZnO NPs using *R. fairholmianus* root extract and their antibacterial effects [29]. To our knowledge, no study assessing the in vitro anticancer effects of ZnO NPs capped with *R. fairholmianus* root extract in MCF-7 breast cancer cells has been reported. The anticancer potential of RFZnO NPs in MCF-7 cells was determined by analyzing cytotoxicity, proliferation, reactive oxygen species (ROS), and apoptotic cell death.

## 2. Results

### 2.1. Morphological Assessment, Cell Proliferation, and Cytotoxicity Analysis

**Figure 1:**
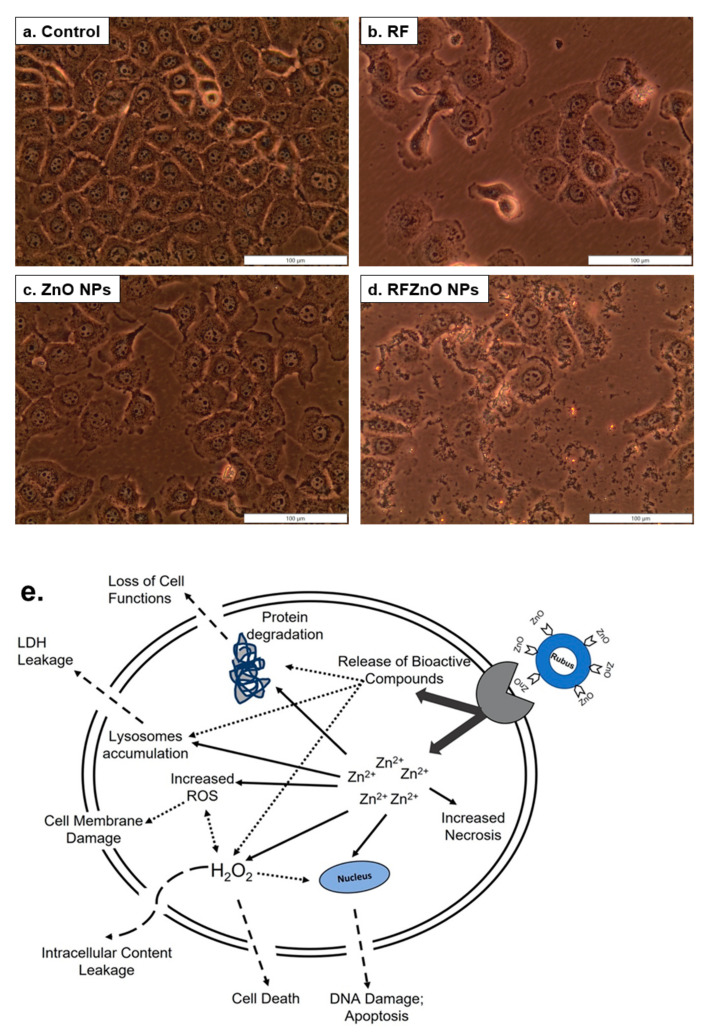
Morphological changes in MCF-7 breast cancer cells 24 h after treatment with *Rubus fairholmianus* (RF 10 µg/mL), zinc oxide nanoparticles (ZnO NPs 10 µg/mL), and *R. fairholmianus* capped zinc oxide nanoparticles (RFZnO NPs 10 µg/mL). Control cells did not show any sign of cell death and were found to be healthy and confluent (**a**). Treated groups showed more dead cells with loss of membrane integrity. An increased number of dead cells was seen in RF (**b**) and RFZnO NP (**d**) treatment groups compared to the control group (**a**) and cells treated with ZnO NP (**c**). (**e**) Possible cell death/cytotoxicity mechanism of action induced by RFZnO NPs.

**Figure 2:**
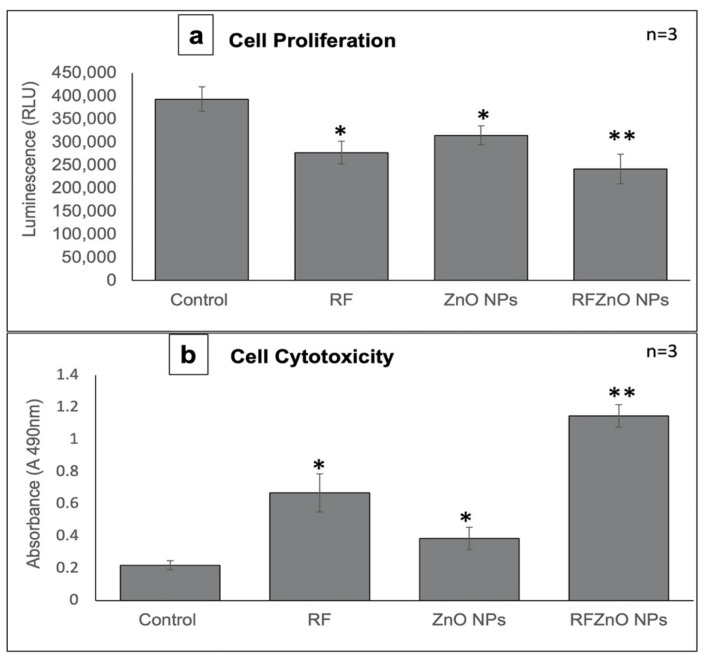
(**a**) Measurement of cell proliferation by adenosine triphosphate (ATP) luminescent assay. Control cells showed maximum levels of ATP. ATP levels were significantly decreased in cells treated with Rubus fairholmianus (RF 10 µg/mL), zinc oxide nanoparticles (ZnO NPs 10 µg/mL), and R. fairholmianus capped zinc oxide nanoparticles (RFZnO NPs 10 µg/mL). (**b**) Lactate dehydrogenase (LDH) was used to measure cytotoxicity. A significant increase in cytotoxicity 24  h after treatment with RF, ZnO NPs, and RFZnO NPs was observed. Significant differences are shown as * *p* <  0.05 and ** *p* < 0.01. Results represent the mean ± SE of three independent experiments.

The changes in the shape of treated and untreated breast cancer cells were analyzed. As compared to healthy untreated breast cancer cells, treated cells showed significant morphological variations such as irregular shape and rounding, as well as detachment from the culture plate and decreased confluency. The RF- and RFZnO-treated cells showed significant morphological changes compared to ZnO NP-treated and untreated cells (Figure 1a–e). The proliferation of breast cancer cells following the treatments was evaluated using the adenosine triphosphate (ATP) proliferation assay. Cellular proliferation in control cells was higher, as was evident from the increased ATP content. Exposure of cells to RF, ZnO NPs, and RFZnO NPs led to a reduction in intracellular ATP levels, which indicates reduced cellular proliferation. Cells incubated with RFZnO NPs resulted in a significant decrease (*p* < 0.01) (Figure 2a) in cell proliferation compared to untreated cells. The cytotoxic effects of RF, ZnO NPs, and RFZnO NPs were measured using the lactate dehydrogenase (LDH) assay. The release of LDH from damaged cells (through a ruptured cell membrane) into the culture media is a direct measure of cellular toxicity. Untreated MCF-7 cells showed the least amount of LDH release (absorbance: 0.2183) compared to RF- and ZnO-treated cells (absorbance: 0.361 and 0.616, respectively). A significant 5.22-fold (*p* < 0.01) increase in toxicity (absorbance: 1.141) was observed in RFZnO NP-treated cells (Figure 2b). These results indicate that the NPs synthesized from *R. fairholmianus* decreased cellular proliferation and increased cytotoxicity in MCF-7 cells 24 h post-treatment.

### 2.2. Cytochrome C Release and Caspase 3/7 Activity

**Figure 3:**
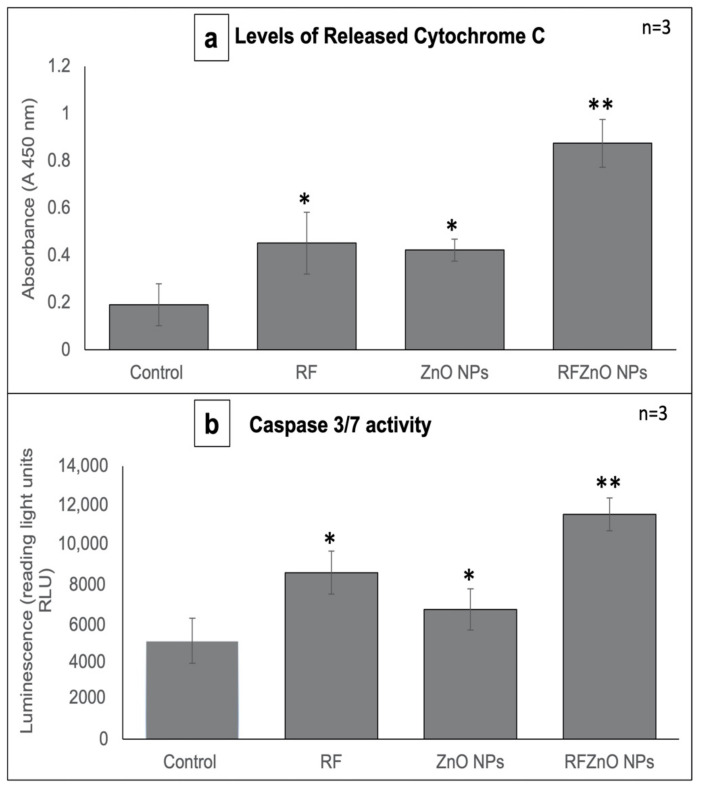
(**a**) Cytochrome c levels in control and treated MCF-7 cells. A significant increase in cytochrome c levels was observed in all treated cells when compared with the control. (**b**) Caspase 3/7 activity. A significant increase in caspase 3/7 activity was observed after treatments. Significant differences are shown as * *p*  <  0.05 and ** *p* < 0.01. Results represent the mean ± SE of three independent experiments.

Cytochrome C (cyt c) is a water-soluble hemeprotein (~12 kDa) mainly located in the inner mitochondrial membrane. Mitochondrial release of cytotochrome c into the cytoplasm is a vital process in the execution phase of apoptosis. Cyt c functions as an electron transporter/vehicle in the respiratory chain and is then translocated to the cytosol in cells that undergo apoptosis, where it is involved in the activation of the caspase cascade signaling pathway [30]. Many apoptotic proteins induce mitochondrial damage resulting in the release of cyt c and thereby the activation of caspases. The cyt c assay results reveal that compared to the untreated cells, a significant amount of cyt c was released by treated cells (Figure 3a). A significant (*p* < 0.01) increase in cyt c release was observed in RFZnO NP-treated cells compared to untreated cells with a 4.57-fold increase. RF- and ZnO NP-treated cells showed a 2.36- and 2.20-fold increase in cyt c release, respectively.

Previous studies have documented that caspase 3 is important in regulating DNA fragmentation and the initiation of apoptosis, whereas caspase 7 plays a major role in cell cycle progression and tumor growth. Based on these facts, we determined the levels of caspase 3/7 as a measure of apoptosis [31] 24 h after treatment with RF, ZnO NPs, and RFZnO NPs using the Caspase-Glo 3/7 luminescent assay kit. Caspase 3/7 levels were compared with untreated cells, and the results revealed that RFZnO NP treatment induced a noticeable increase (*p* < 0.01) in caspase 3/7 (Figure 3b).

### 2.3. Reactive Oxygen Species (ROS) Production and Nuclear Hoechst Staining

**Figure 4:**
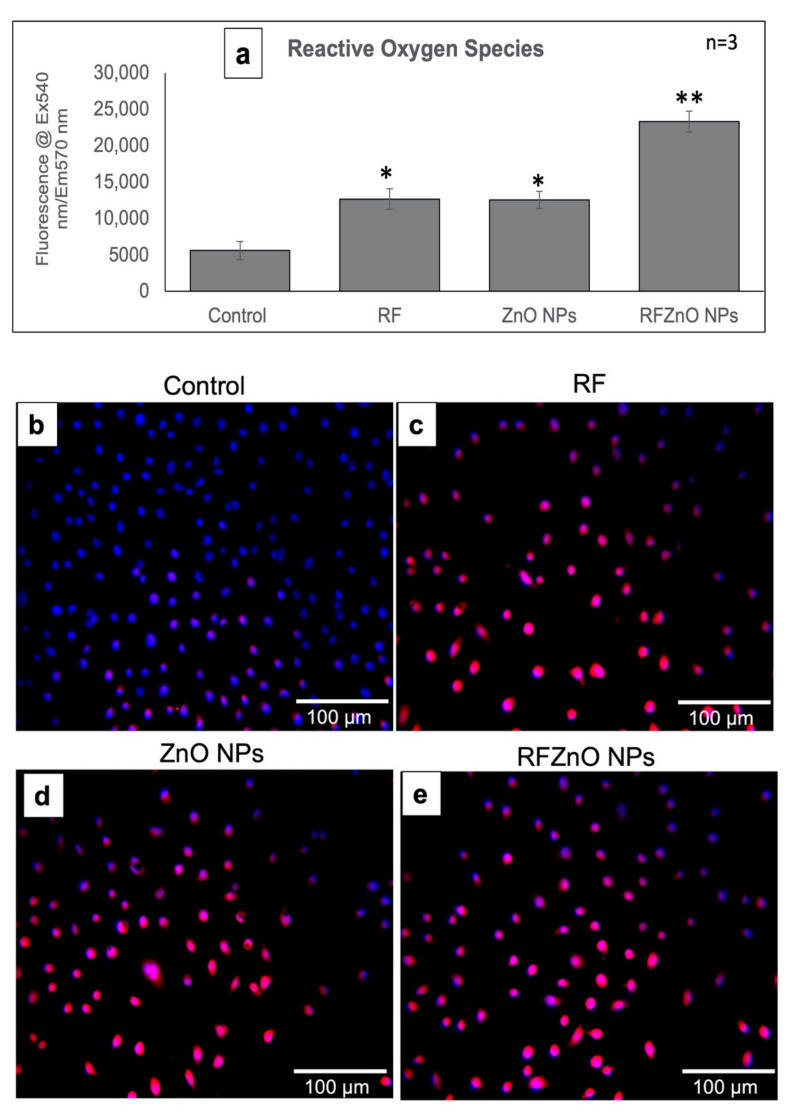

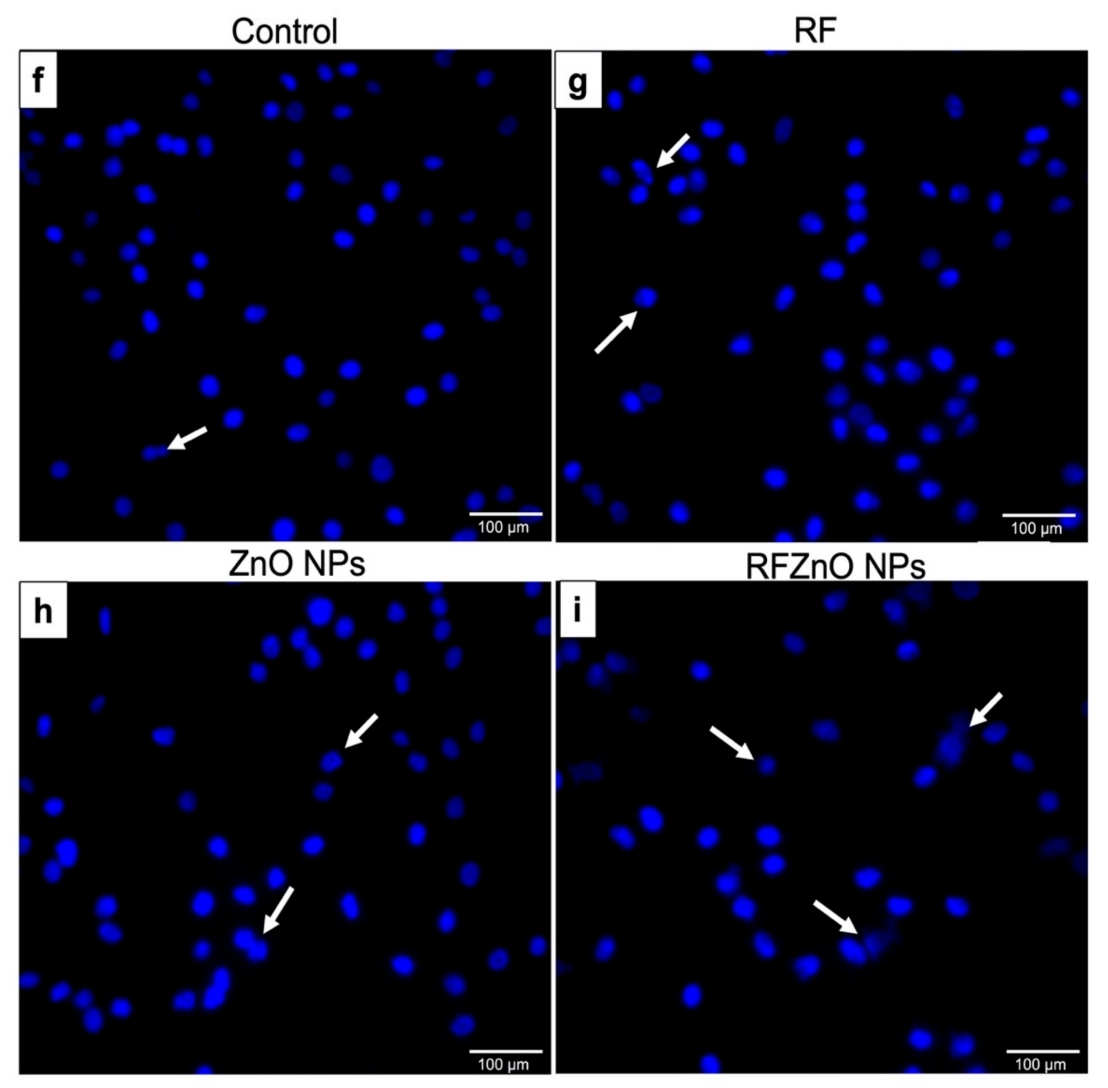
(**a**) Measurement of reactive oxygen species (ROS) in control and treated MCF-7 cells. There was a significant increase in ROS in *R. fairholmianus* capped zinc oxide nanoparticle (RFZnO NP)-treated groups. Significant differences are shown as * *p*  <  0.05 and ** *p* < 0.01. Results represent the mean ± SE of three independent experiments. (**b**–**e**) Qualitative assessment of ROS: ROS are indicated by red fluorescence and nuclei are stained blue. There was increased red fluorescence, and hence increased ROS, in the treatment groups, while in the control group ROS production was lower. (**f**–**i**) Nuclear damage as determined by Hoechst staining. Treatments induced minor nuclear damage in MCF-7 cells; the intensity of nuclear stain was decreased, indicating nuclear disintegration (as shown by the arrows).

ROS play an important role in inducing apoptosis in cancer cells. The production of ROS was evaluated after exposure to RF, ZnO NPs, and RFZnO NPs using ELISA (Figure 4a) and qualitative fluorescence methods (Figure 4b–e), and nuclear damage was assessed qualitatively through Hoechst staining. Increased intracellular ROS levels were observed in the treated groups (RF and ZnO NPs: *p* < 0.05; *RFZnO NPs:* *p* < 0.01) (Figure 4a). The presence of red fluorescent signals in treated groups shows increased levels of ROS (Figure 4c–e). Similarly, groups incubated with RF, ZnO NPs, and RFZnO NPs showed slight nuclear damage as determined by the Hoechst stain. Untreated cells presented with homogeneously stained round nuclei, while treated cells displayed an uneven nuclear shape and DNA condensation (Figure 4f–i). These results reflect that RFZnO NPs can induce cytosolic oxidative stress and cell death.

### 2.4. Immunofluorescence and Immunoblotting of p53, Bax, and Bcl-2

**Figure 5:**
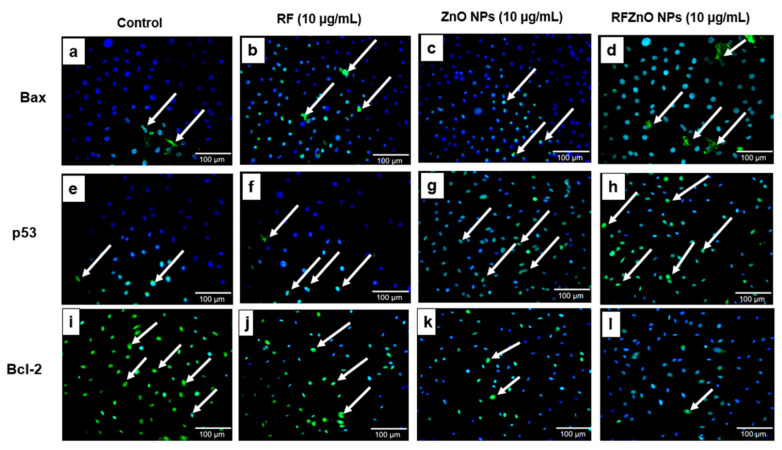
Immunofluorescence images of Bax (**a**–**d**), p53 (**e**–**h**), and Bcl-2 (**i**–**l**) in control and treated cells. Treated cells showed significant changes in the activity of apoptotic proteins. Control cells showed few p53- and Bax-positive cells and more Bcl-2-positive cells. Cells treated with Rubus fairholmianus (RF), zinc oxide nanoparticles (ZnO NPs), and R. fairholmianus capped zinc oxide nanoparticles (RFZnO NPs) showed higher levels of p53 and Bax positivity. The treatment with RF, ZnO NPs, and RFZnO NPs decreased Bcl-2. Magnification: 20X original; arrows indicate the presence of proteins p53, Bax, and Bcl-2 (FITC stained, green); nuclear stain: DAPI (blue).

Apoptotic proteins were studied to determine the influence of RF, ZnO NPs, and RFZnO NPs in inducing the apoptotic pathway in breast cancer cells. From the results, apoptotic proteins Bax (Figure 5a–d) and p53 (Figure 5e–h) were found at high levels in treated groups, specifically in RFZnO NP-treated cells, while the anti-apoptotic protein Bcl-2 (Figure 5i–l) was reduced considerably in the treated groups. Following treatment, we observed activated p53 nuclear translocation, and hence more p53 was observed in the nucleus while the Bax was seen in the cytosol. Arrows in the figure indicate the presence of p53, Bax, and Bcl-2 proteins.

**Figure 6:**
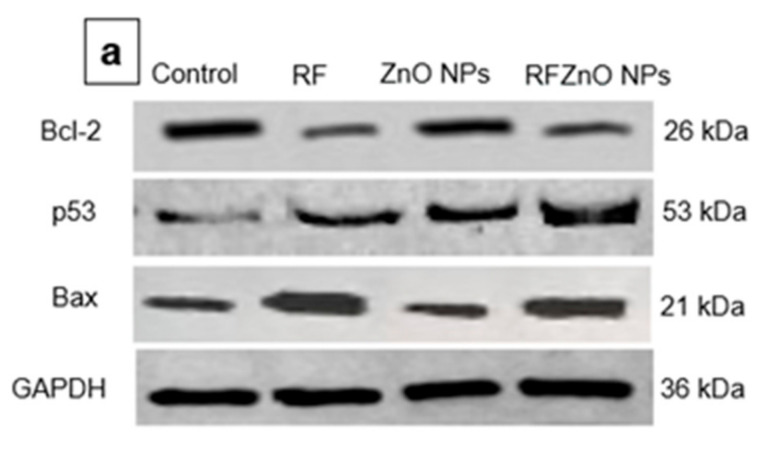

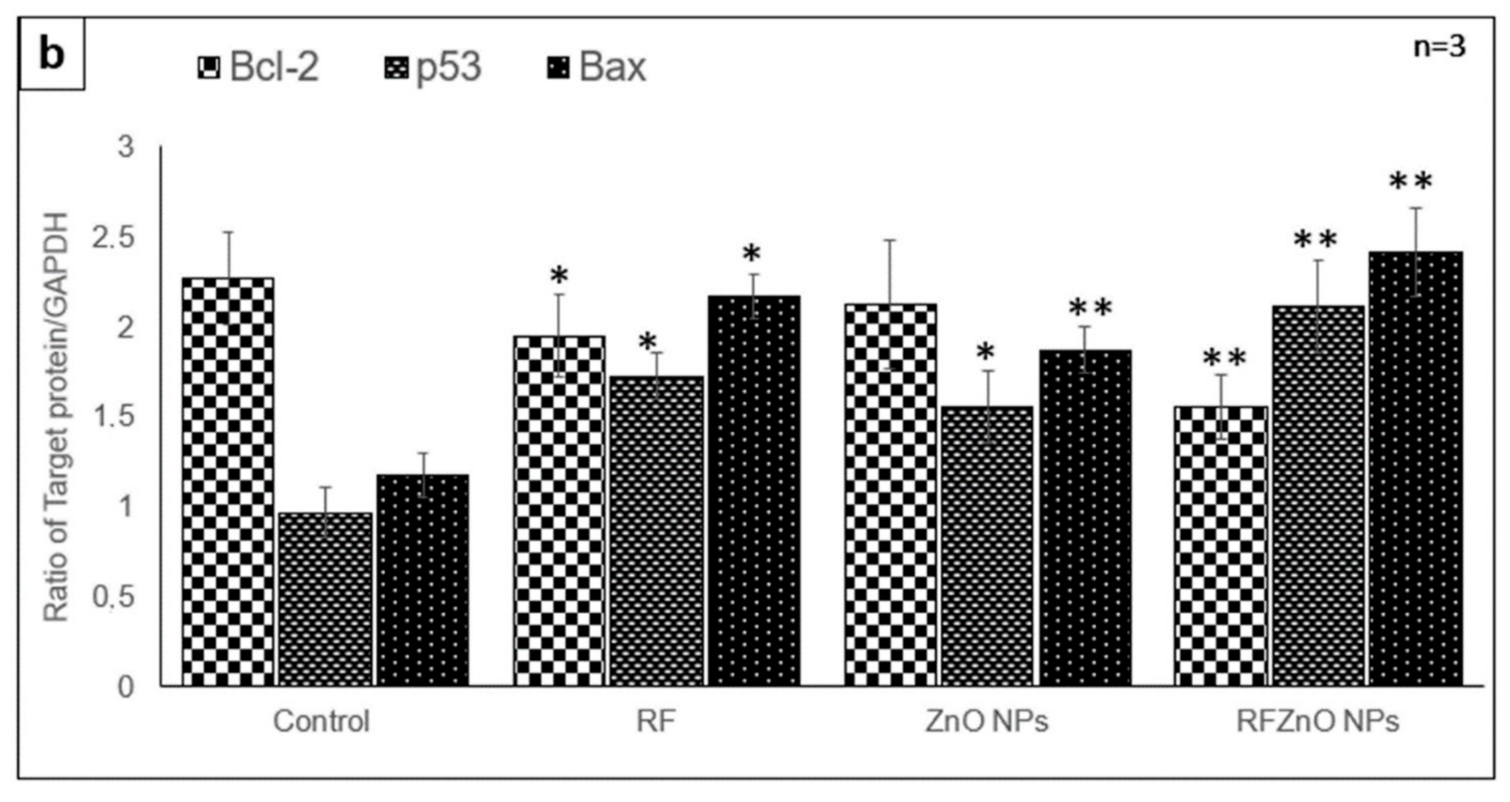
Effect of RFZnO NPs on the level of p53, Bax, and Bcl-2 apoptotic proteins, as determined by immunoblotting (**a**). L1: control; L2: *Rubus fairholmianus* (RF) (10 µg/mL); L3: zinc oxide nanoparticles (ZnO NPs) (10 µg/mL); L4: *R. fairholmianus* capped zinc oxide nanoparticles (RFZnO NPs) (10 µg/mL). The results showed that p53 and Bax levels were significantly increased, while the level of Bcl-2 was decreased in treated groups. Relative expression shown in a graph (**b**). Protein levels were quantified using ImageJ software and normalized to GAPDH band intensity. Significant differences are shown as * *p*  <  0.05 and ** *p* < 0.01. Results represent the mean ± SE of three independent experiments.

We also examined the level of these proteins by Western blotting (Figure 6a,b) and observed a similar pattern. From the immunoblot results, the levels of Bax and p53 pro-apoptotic proteins were high in cells treated with RFZnO NPs, while the level of anti-apoptotic protein Bcl-2 appeared to be low. The RFZnO NP-treated groups showed significantly increased levels (*p* < 0.01) of Bax and p53, while reduced levels (*p* < 0.01) of Bcl-2 were observed. The activation of p53 and Bax leads to the loss of mitochondrial membrane potential and cyt c release, and immunofluorescence assays also support these findings.

## 3. Discussion

ZnO NPs are multi-faceted metal oxide NPs with diverse biological properties [32]. This study investigated the anticancer activity of green synthesized ZnO NPs against breast cancer cells. As revealed by morphological analysis by inverted microscopy, the RFZnO NP-treated MCF-7 cells showed significant morphological variations, including rounding of cells and detachment from culture plates. In addition, the ATP proliferation results displayed a significant (*p* < 0.01) decrease in proliferation after RFZnO NP treatment. However, RF- and ZnO NP-treated groups also showed decreased proliferation (*p* < 0 05). The LDH cytotoxicity assay results were supportive of the morphology and proliferation assay results. RF, ZnO NP, and RFZnO NP treatment significantly (*p* < 0.01) induced cytotoxicity by releasing LDH into the culture media. Cytoplasmic LDH will be released into the cell culture media upon the loss of cell membrane integrity. The cytotoxicity results revealed that there is an increased cell death observed in the treated groups. This might be due to the synergistic action of *R. fairholmianus* and ZnO NPs, and it can be postulated that when cells are treated with RFZnO NPs, there will be an accumulation of Zn^2+^ in the cytosol [33] and the *Rubus* bioactive compounds will be released into the cytosol, which will result in an increased free radical production and oxidative stress which in turn might induce DNA and cell membrane damage and loss of cellular function [25,26]. The released bioactive compounds also activate apoptotic signals, resulting in cell death (Figure 1e). LDH activity is a direct measure of cellular toxicity and cell death due to cell membrane rupture and apoptosis [34]. Similarly, the cell proliferation biomarker ATP is abundant in all metabolically active viable dividing cells. Higher ATP levels indicate increased cell proliferation rates. The reduction in ATP levels is directly linked with reduced cell proliferation [35]. In this study, the data from cell morphology analysis, cytotoxicity, and proliferation assays provided evidence for the reduction in cell numbers in treated groups. Reports show that ZnO NPs induce structural changes including the loss of cell-to-cell adhesion in human cervical carcinoma cells [36].

Cyt c is a vital mitochondrial peripheral protein, and it acts as an electron shuttle and contributes to apoptosis. The intermembrane mitochondrial space (IMS) comprises a heterogeneous group of proteins that promote cell death upon release. The first molecule belonging to this group of proteins is cyt c. Upon apoptotic stimuli, cyt c is released into the cytoplasm where it activates the adaptor molecule in the presence of ATP and generates a complex known as the apoptosome to execute apoptotic cell death [37,38]. Previous studies reported that ethanolic extracts of medicinal plants induce apoptosis via intrinsic and extrinsic pathways. The loss of mitochondrial membrane potential is a major characteristic of mitochondria-dependent apoptosis [39,40]. Hence, the analysis of mitochondrial cyt c release is an important measure of apoptotic cell death. The results (Figure 3a) show that RFZnO NPs significantly induced cyt c release (*p* < 0.01).

Apoptotic events are strongly controlled by numerous caspase proteins that ultimately mediate cell death. Caspase 3 activation occurs in the perinuclear area of the cell and is associated with mitochondrial cyt c release which can be prevented by the upregulation of Bcl-2 proteins [41]. McComb et al. reported that the stimulation of caspase 3 or 7 initiates the augmentation of the upstream apoptotic process, which is a vital feature of cell death via apoptosis. The activity of caspase 3 or 7 is essential for mitochondrial membrane depolarization, and hence cyt c release [42]. Results from this study showed that a 2-fold increase in caspase 3/7 activity was obtained (Figure 3b) after treatment with RFZnO NPs (*p* < 0.01).

Even though higher levels of ROS play a vital role in cancer incidence and progression, levels above a cytotoxic threshold lead to cancer cell death. Many commonly used anticancer therapies such as radiation, chemotherapy, and photodynamic therapy (PDT) depend on ROS production to induce cancer cell death mechanisms. Numerous reports proved that ZnO NPs exert significant antitumor activity via the generation of ROS and free radical production, and this is regarded as one of the pathways by which ZnO NPs induce cancer cell death [42,43]. Increased ROS levels lead to a decrease in antioxidant enzymes and ultimately result in cellular oxidative damage [44]. It is well proven that ROS can regulate the translocation, phosphorylation, and/or cleavage of pro-apoptotic Bcl-2 family proteins, leading to apoptosis induction [45]. Our quantitative (ELISA) (Figure 4a) and qualitative (immunofluorescence) (Figure 4b–e) experimental results showed the increased production of ROS following treatments (*p* < 0.01). Nuclear damage was assessed by Hoechst staining, which did not indicate significant nuclear damage after treatments; the control and treated cells looked similar, although minor nuclear damage was seen in treated cells (Figure 4f–i).

Further, we studied the cell death mechanism caused by ZnO NPs and RFZnO NPs using immunofluorescence and immunoblot analysis of vital apoptotic proteins (Bax, Bcl-2, and p53). Levels of pro-apoptotic proteins (Bax and p53) were increased, whereas the anti-apoptotic Bcl-2 activity was reduced. Mitochondria-mediated apoptosis requires mitochondrial membrane depolarization to release apoptotic mediators such as cyt c [38]. Tumor suppressor gene P53 is involved in the mediation of apoptosis via the regulation of BCL-2 family genes [46]. The Bcl-2 family proteins involve pro-apoptotic and anti-apoptotic members. Bcl-2 proteins are vital mediators which control the mitochondrial pores to regulate cyt c release to aid in the apoptotic process [38,39,40]. The anti-apoptotic Bcl-2 proteins help in cell survival by inhibiting the pro-apoptotic proteins [40]. Bax controls cell death by disrupting the mitochondria, and its activity is in turn controlled by p53 [42]. The overexpression of Bax improves mitochondrial pore opening followed by cyt c release [47]. Several studies showed that translocation of Bax without Bax/Bcl-2 ratio variation can upregulate caspase 3 to execute apoptosis [48,49,50]. Similar to the findings of Akhtar et al. [51] in human lung cancer cells (HepG2), our results (breast cancer cells) also reported significantly increased levels of p53 and Bax in treated cells, while there were reduced levels of Bcl-2.

**Figure 7:**
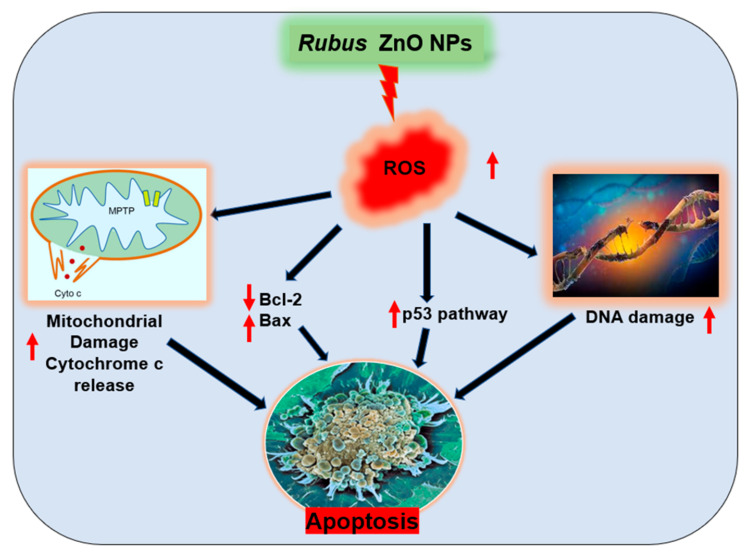
Proposed cell death mechanism induced by *Rubus fairholmianus* capped zinc oxide nanoparticles (RFZnO NPs). The reactive oxygen species (ROS) produced during RFZnO NP treatment induced mitochondrial cytochrome c release, induced nuclear damage, and increased the activity of p53 and Bax while reducing the activity of Bcl-2, leading to apoptotic cell death in breast cancer cells.

The results presented show that RFZnO NPs induce p53 and Bax (pro-apoptotic); however, they did not induce anti-apoptotic Bcl-2. Furthermore, treatment with RFZnO NPs also leads to the activation of caspase 3/7, suggesting that a mitochondrial apoptotic cell death mechanism is involved in MCF-7 breast cancer. These results are also in correlation with the ROS analysis. Mohammadinejad et al. [52] reported that several kinds of oxidative stress activate intrinsic apoptosis. Our data also showed that RFZnO NPs enhanced intracellular ROS levels, and subsequently disrupted mitochondrial function by the release of cyt c in breast cancer cells. Hence, we propose that RFZnO NP-induced apoptosis might have been caused by oxidative stress in breast cancer cells as shown in the proposed mechanism (Figure 7).

## 4. Materials and Methods

### 4.1. Plant Material, Collection Site, and Herbarium

*Rubus fairholmianus* Gard. was collected from Munnar, Kerala, India, and a herbarium sample BSI/SRC/5/23/2010-11/Tech.1657 was deposited in the Botanical Survey of India.

### 4.2. Extraction and Green Synthesis of ZnO NPs

The freshly collected root of *R. fairholmianus* was cleaned and shade dried. The powdered roots were used for the hot percolation Soxhlet extraction process using acetone. About 200 mg of root acetone extract was dissolved in 10 mL of 0.5% DMSO for in vitro studies.

Green synthesis of ZnO NPs: Previously we have reported on the green synthesis of ZnO NPs using R. fairholmianus root extract. The protocol explained by Rajendran et al. [29] using zinc nitrate was employed in the synthesis of ZnO NPs.

### 4.3. Cell Culture

Human breast adenocarcinoma cells (MCF-7, ATCC HTB-22) were used in this study. Dulbecco’s modified Eagle medium (DMEM) was used for cell culture with 10% fetal bovine serum (FBS; Gibco 306.00301), 1% penicillin/streptomycin (PAA Laboratories GmbH, P11-010), and 1 µg/mL amphotericin B (PAA Laboratories GmbH, P11-001). Cells were grown at 37 °C, 5% CO_2,_ and 80% humidity in a CO_2_ incubator. Hank’s Balanced Salt Solution (HBSS, Merck, Johannesburg, South Africa) was used to wash cells when confluent. Cells were detached using 1 mL/cm^2^ TryplExpress (Gibco, ThermoFischer Scientific, 12604, Johannesburg, South Africa) for subculturing. For experiments, 5 × 10^5^ cells were seeded in 3.5 cm diameter cell culture plates, allowed to attach for 6 h, and treated/incubated for 24 h with 10 µg/mL of RF, ZnO NPs, and RFZnO NPs for each experiment.

### 4.4. Morphology, Cell Proliferation, and Cytotoxicity Analysis

The morphological variations in cells after 24 h incubation with newly synthesized nanomaterials were observed using cellSens imaging software and an Olympus CKX 41 inverted light microscope (Wirsam, Johannesburg, South Africa). Treated cells were washed with HBSS before images were captured.

The CellTiter-Glo Luminescent cell viability assay (Promega, G7571, Anatech Analytical Technology, Johannesburg, South Africa) was used for the quantitative analysis of metabolic ATP contents in living cells. About 50  µL of ATP reagent and 50  µL cell suspension were incubated in the dark for 10  min at room temperature before luminescence was measured (in relative light units, RLU) on a Victor3 1420 Multilabel Counter (Perkin-Elmer, Separation Scientific, Johannesburg, South Africa). The estimation of ATP levels is a measure of cell proliferation. The CytoTox 96 Non-Radioactive Cytotoxicity Assay kit (Promega G 400, Anatech Analytical Technology, South Africa) was used to assess the cytotoxic activity of green synthesized NPs. Lactate dehydrogenase (LDH) released into the media measures the membrane integrity of the cells after treatment, which indirectly corresponds to cytotoxicity. Equal volumes (50  µL) of LDH reagent and culture medium were incubated for 30 min in the dark at room temperature and measured at 490  nm (Victor3 1420 Multilabel Counter, Perkin-Elmer, Separation Scientific).

### 4.5. Cytochrome C Release and Caspase 3/7 Assay

Cytochrome c is a vital factor for apoptotic events. Cytochrome c (cytoplasmic cyt c) levels were measured using the human cytochrome c Platinum ELISA assay kit (Invitrogen, KH01051, ThermoFisher Scientific, Johannesburg, South Africa), and the downstream executioner caspase 3/7 activities were measured by the Caspase-Glo 3/7 assay (Promega G8091, Anatech Analytical Technology, South Africa) according to the manufacturer’s protocol [27].

### 4.6. Hoechst Staining and Reactive Oxygen Species (ROS) Production

ZnO NP-induced nuclear damage was studied by Hoechst nuclear staining (Hoechst 33258, H21491, Sigma-Aldrich, Johannesburg, South Africa) as explained by George et al. [19]. ROS formed in response to treatment with NPs and plant extract were analyzed using the dichlorodihydrofluorescein diacetate (DCFH-DA) assay [27]. In brief, for quantitative measurement of ROS, cells were grown in 96-well plates, and once they reached 80 to 90% confluency, they were treated for 24 h with different treatment solutions at a concentration of 10 µg/mL. After the treatment period, the medium was removed and cells were incubated with 100 µM of DCFH-DA in 100 µL of HBSS for 30 min at 37 °C. ROS generated in the cells were immediately measured at Ex: 540 nm, Em: 570 nm using the Victor3 1420 Multilabel Counter (Perkin-Elmer, Separation Scientific, South Africa). For the staining of ROS, the cells were grown on sterile coverslips in a clear-bottom 6-well plate. Once cells reached 80% confluency, they were treated similarly, i.e., 10 µg/mL treatment solutions for 24 h. After treatment, the media were replaced with fresh media and treated with 100  μM DCFH-DA for 30  min in the dark. Cells were washed once with phosphate-buffered saline (PBS) and stained with 1 µg/mL of nuclear stain (DAPI, Invitrogen, D1306, Sigma-Aldrich, South Africa). Finally, the coverslips were washed with PBS and fixed on a glass slide. Image were captured with AxioVision imaging software (Version 4) installed on a Carl Zeiss Axio Observer Z1.

### 4.7. Immunofluorescence and Immunoblotting

The qualitative and quantitative levels of apoptotic proteins (p53, Bax, and Bcl-2) after treatment with RFZnO NPs were determined using immunofluorescence and immunoblotting, respectively, as previously explained by George et al. [27]. The cells were cultured on coverslips for immunofluorescence experiments, whereas for Western blotting the proteins were isolated 24 h post-treatment, separated by SDS PAGE, and transferred to a PVDF membrane. We used the primary antibodies p53 (Pab 240) (mouse monoclonal antibody, Santa Cruz Biotechnology SC-99, Anatech, Johannesburg, South Africa) Bax monoclonal antibody (2D2) (mouse monoclonal antibody, Life Technologies 336400, ThermoFisher Scientific, Johannesburg, South Africa), Bcl-2 monoclonal antibody (Bcl-2-100) (Life Technologies 13-8800), and GAPDH loading control monoclonal antibody (GA1R) (mouse monoclonal antibody, Invitrogen MA5-15738), and we used a horseradish peroxidase-conjugated secondary antibody (goat anti-mouse HRP, Santa Cruz Biotechnology SC-2005) for Western blotting and a goat anti-mouse IgG (H+L) Superclonal recombinant secondary antibody, Alexa Fluor 488 (ThermoFisher Scientific A28175), for immunofluorescence.

### 4.8. Statistical Analysis

The data are represented as the standard error of the mean (SEM) of three replicates (n = 3) performed in duplicate. The statistical significance was analyzed using SigmaPlot version 14.0. Untreated control cells were used to compare the statistical significance of treated cells. The differences between groups were determined using paired Student’s *t* test and one-way analysis of variance (ANOVA). A *p* value less than 0.05 was considered significant.

## 5. Conclusions

In this study, we established that RFZnO NPs can significantly induce cytotoxicity in breast cancer cells via the induction of apoptosis and ROS production. The effect of RFZnO NPs could be directly linked with mitochondrial cyt c release to induce intrinsic apoptosis. Moreover, RFZnO NPs could increase the levels of pro-apoptotic proteins Bax and p53, eventually leading to apoptosis. Hence, the results of this study lead to the conclusion that apoptosis induction is directly linked with the toxicity of RFZnO NPs through ROS generation, increased levels of apoptotic proteins, and the release of the mitochondrial marker cyt c. The mechanisms behind the activity of RFZnO NPs necessitate an additional detailed study to validate the cell death pathway by protein and gene expression profiling.

## Data Availability

Not applicable.
